# How does lead induce male infertility?

**Published:** 2011

**Authors:** Mohsen Vigeh, Derek R. Smith, Ping-Chi Hsu

**Affiliations:** 1 Department of Hazard Evaluation and Epidemiology Research, National Institute of Occupational Safety and Health, Kawasaki, Japan.; 2 School of Health Sciences, Faculty of Health, University of Newcastle, Ourimbah, Australia.; 3 Departmant of Safety, Health and Environmental Engineering, National Kaohsiung First University of Science and Technology, Kaohsiung, Taiwan.

**Keywords:** *Lead*, *Male reproduction*, *Infertility*, *Reproductivehormone*, *Spermatogenesis*

## Abstract

An important part of male infertility of unknown etiology may be attributed to various environmental and occupational exposures to toxic substances, such as lead. The reproductive effects of lead are complex and appear to involve multiple pathways, not all of which are fully understood. It is still unclear, for example, if male reproductive issues in lead-exposed persons are mostly related to the disruption of reproductive hormones, whether the problems are due to the lead’s direct effects on the gonads, or both? This question has been difficult to answer, because lead, especially at high levels, may adversely affect many human organs. Although lead can potentially reduce male fertility by decreasing sperm count and motility, inducing abnormal morphology and affecting functional parameters; not all studies have been able to clearly demonstrate such findings. In addition, research has shown that the blood-testis barrier can protect testicular cells from direct exposure to high levels of blood lead. For these reasons and considering the wide spectrum of lead toxicity on reproductive hormones, the present review suggests that lead’s main influence on male reproduction probably occurs by altering the reproductive hormonal axis and the hormonal control on spermatogenesis, rather than by a direct toxic effect on the seminiferous tubules of the testes. As blood lead concentrations below the currently accepted worker protection standard may still adversely affect male fertility, future studies should aim to establish more concrete links between lead exposure (especially at low levels) and subsequent male infertility. Research should also pay more attention to lead’s effects on reducing male fertility rates based on not only hormonal axis alteration, but also on the changes in sperm characteristic among exposed subjects.

## Introduction

Many industrial chemicals are known to have a negative impact on human reproduction (1-3), particularly occupational and environmental exposures to heavy metals such as lead (3-8). The risk is generally believed to be directly correlated with both increasing concentrations and duration of exposure (9, 10). At its simplest, increased blood lead levels of 12.5μg/dl versus 6.0μg/dl, have been observed among infertile men when compared to fertile men, respectively (11). Similarly, epidemiological studies on male workers with blood lead levels ranging from 10 to more than 40µg/dl have been shown to increase the risk of infertility (6, 12). A study of more than 4000 male workers with blood lead levels higher than 25μg/dl, for example, demonstrated a reduction in the number children when compared to 5000 control subjects (13).

 Experimental animal studies, mainly in rats, have also reported that lead is an active element responsible for male reproductive parameter imbalances (14, 15). On the other hand, a multi-country (Belgium, Finland, Italy, and England) investigation found no association between occupational exposure to lead and lower fertility rates when blood lead concentrations ranged from 29 to 37μg/dl (16). In addition, studies on lead battery workers were unable to confirm the effects of lead on male fertility among French (17), Danish (18) and Taiwanese workers with blood lead concentrations of ≤29μg/dl (19). Although the evidence is not conclusive, a threshold for adverse reproductive effects in men might be in the blood lead range of around 30 to 40μg/dl (20). Nevertheless, lead’s adverse effects on male reproductive function, particularly at low levels (<10μg/dl), has still not been adequately reviewed. Approximately 15% of couples attempting their first pregnancy meet with failure (21), and contemporary data suggests that male-related factors are responsible for around half of all infertility cases (22). 

Because human sperm count, normal morphology and functions appear to be in decline (a situation that may potentially jeopardize male fertility) increasing attention has been paid to male reproductive problems in recent years (23). On the other hand, an important component of male infertility of unknown etiology may be attributable to environmental and occupational to various chemical exposures (2, 24). In this article, we provide an overview of epidemiological and experimental studies published in English up to December 2009, available on PubMed (U.S. National Library of Medicine) and that addressed lead toxicity on the male reproductive system. The article is divided into three parts; 1) Spermatogenesis, 2) Sperm functional parameters, and 3), Hormonal disruptions. Our review also elucidates the most likely responsible mechanism of lead on the male reproductive system, as this is still not clearly understood.

**Spermatogenesis**

The most frequent causes of male infertility are associated with spermatogenesis. Because it is relatively easy to conduct, non-invasive and inexpensive to perform, semen analysis (sperm count, semen volume, sperm morphology and assessments of functional parameters) is one of the first laboratory tests commonly performed for infertile couples. Studies on occupationally lead-exposed men have shown multiple sperm parameters being affected as seminal plasma or blood lead concentrations rise, usually at levels of >40μg/dl, but sometimes even at levels of <10μg/dl. For instance, reductions in sperm count and sperm concentration or density (5, 10, 25, 26), decreased volume of ejaculation (10, 26), as well as correlations with asthenospermia, hypospermia, and teratospermia (53μg/dl) (27) have been reported in male workers. Furthermore, higher percentages of immature and abnormal spermatozoa such as wide, round, and short sperm in lead exposed workers have been reported at both high (40μg/dl) and low (<15μg/dl) blood lead levels (28-30). 

Many studies on reproductive system of male animals have documented lead as a toxicant for testicular tissue and functions (31-34) such as significant reductions in the number of spermatozoa within the epididymis in mice administered lead acetate (0.25% and 0.50%) in drinking water (35) and halted spermatogenesis in rats (36). Many studies suggest spermatogenesis problems caused by lead, although, some researchers have failed to demonstrate correlations between lead and semen volume, pathologic sperm and sperm concentration among workers exposed to high lead levels (27, 37), or abnormalities in sperm count and/or the sperm morphology in rabbits (38). 

Macroscopic changes in accessory sex organs such as diminished weight of testes, seminal vesicles, epididymis, and ventral prostate have been demonstrated in various studies using experimental animals (39, 40). Microscopic changes, histological as well as macroscopic ones, have been induced by increasing lead levels in lead exposed male rats (41, 42) including changes in the testicular tissues morphology (36, 43), and decreased germ cells layer population (36, 42). In addition, two studies conducted on lead exposed mice demonstrated seminiferous tubule degeneration (44), and seminal abnormal cytology (45). Similarly, electron microscopic analysis has revealed that lead-exposed monkeys, when exposed during infancy, can induce testicular alterations, which persist in later life even when blood lead concentrations had decreased considerably (46). Due to ethical limitations, many studies on reproductive organs have been performed using high lead levels with experimental animals, which have revealed lead’s effect at the cellular level.

**Sperm functional parameters**

Successful fertilization of an ovum by spermatozoa depends not only on sperm count and morphology but is also relevant to functional parameters. Lead has been shown to incur detectable negative effects on blood, semen and/or spermatozoa quality in workers, such as inducing prolonged liquefaction time and decreasing sperm motility (26). It has been negatively associated with sperm motility and viability (blood lead levels ≤ 10 µg/dl) (47), and a reduction in the functional maturity of sperm among men with mean blood lead levels of 45µg/dl (48). 

On the other hand, concomitantly, significant improvements in the number of motile sperm has been reported after mean blood lead decreased from 42 µg/dl to 20 µg/dl among the lead factories workers (49). Reduced semen quality such as prolonged latency of semen melting have also been reported amongst lead exposed workers, without directly measuring blood and/or semen lead concentrations (50, 51). As a general rule however, numerous studies have demonstrated that sperm functional disorders induced by lead, are related to the sperm’s interactions with oocytes and implantation, such as premature acrosome loss (52), and strong negative correlation between seminal plasma lead levels and artificial insemination rates in humans (53). 

Two studies of mice and one of rats, have shown that there may be a dose-dependent decrease in the number of sperm attaching to ova (54), reducing the ability of spermatozoa to penetrate the corona radiata and the zona pellucida of the oocyte (55), and an increased frequency of post-implantation loss of embryos (56) (at 0-2 µg/ml lead acetate, 40 µg/dl blood lead levels and 25-50 mg/kg in chow, respectively). According to these results, lead significantly induces the sperm function disorders in exposure cases before and after ejaculation.

**Hormonal disruption**

Reproductive hormones play an important and complicated role in the regulation of spermatogenesis and sperm development. The results of experimental studies in rats have shown that the effects of lead involve multiple sites on male reproductive hormones although the most important part of these disorders probably occurs in the hypothalamic-pituitary-testosterone (HPT) axis (40, 57). For example, depending on lead exposure levels and duration, signals within and between the rat’s hypothalamus and pituitary gland appear to be disrupted by lead (58). In a study of lead-exposed rats hyper responsiveness to stimulation with both gonadotropin releasing hormone (GnRH) and luteinizing hormone (LH) was demonstrated (59). 

Another study on male rats administered lead acetate in water showed a dose-related increase in GnRH mRNA and no effects on the serum concentrations of hypothalamic GnRH or LH, suggesting there may be a compensatory mechanism in the HPT axis (58). In addition to animal experiments, McGregor (1990) reported a positive correlation between serum LH levels and duration of occupational lead exposure (60), a finding which was confirmed one year later in another study of workers with mean blood lead levels of 35µg/dl (61).

Testosterone, the main male sex hormone, is formed and secreted by Leyding cells in testes in response to stimulation by of LH. Semen lead concentrations at a mean of 2 µg/dl have been reported to be inversely related to serum testosterone among occupationally-exposed men (10). 

Suppression of testicular testosterone levels and increasing steroid binding globulin levels related to increased duration of exposure to lead has been also demonstrated among mice exposed to lead for 30 days (62). The suppression of testosterone levels in the epididymal cells and increased androgen binding protein levels of rats have been also noted (15, 40). However, there are some reports describing increased serum testosterone concentrations on lead exposed men from low (median 5 µg/dl) (30) to relatively high (more than 40μg/dl) blood lead levels (63).

These findings suggest that it might involve other hormonal and/or hormonal feedback pathway(s) than disruption of testosterone secretion in the reproductive hormonal axis by lead, such as a lack of reflex in response to plasma testosterone, direct inhibitory androgen biosynthesis in Leydig cells (64), or defects in LH regulation at the pituitary level (57). Molecular mechanisms underlying histopathological examination have revealed disturbance degeneration in Leydig cells among rats (31), thereby suggesting Leydig cells as a target for lead intoxication. 

On the other hand, due to imbalances in the HPT hormonal axis induced by lead exposure, pituitary cells release inappropriate levels of LH and change the steroid negative feedback loop (40), usually at the hypothalamus level (65). Increased concentrations of other reproductive hormones, such as follicle stimulation hormone (FSH), secreted from the pituitary gland, have been observed following lead exposure in men (61) and in lead treated rats (66). However, unchanged concentrations between workers exposed to high and low-levels lead (67) and unmodified levels in mice treated lead with acetate in drinking water (68) have also been shown. These differences in FSH secretion levels might relate to differing lead levels and/or the duration of exposure among subjects. 

On the other hand, inappropriate inhibin B overproduction in excessively lead exposed subjects may be induced by a Cell of Sertoli dysfunction, which suggests spermatogenesis impairment (69). On the other hand, research on male monkeys has shown that alterations in Sertoli cell function may occur due to decreases in inhibin/FSH (70), rather than by a direct effect on the cells. Such findings are consistent with a failure to find significant microscopic alterations in rat’s Sertoli cells, except for increased lysosomal size, verified by ultrastructural examination on the rats’ cells (15, 71). Thus, the Sertoli cells may be not a direct target of lead toxicity and lead’s effects on FSH disruption is the more likely cause of reproductive dysfunction rather than by a direct effect on the cells.

**Mechanisms of lead reproductive toxicity**

At a conceptual level, the mechanisms of lead toxicity on male reproductive system have not yet been fully elucidated. There are a number of probable pathways to explain how lead exposure may reduce male fertility. For instance, multiple calcium and potassium channel isoforms in human testes and spermatozoa, may be involved in early events of acrosome reactions (72). In addition, some enzymes activities, such as alkaline phosphatase and sodium potassium ATPase, have been shown to be reduced in the reproductive organs of lead-exposed rats (36, 73). Another issue in lead’s reproductive toxicity might relate to the excessive generation of *Reactive Oxygen Species* (ROS), an issue which has been paid more attention recently. ROS inhibits the production of sulfhydryl antioxidants, inhibits enzyme reactions, damages nucleic acids and inhibits DNA repair, as well as initiating lipid peroxidation in cellular membranes. Lead induces oxidative stress and promotes the generation of hydrogen peroxide (74, 75). 

The negative wide-ranging of effects due to an increase of ROS levels in tissues have been postulated as a major contributor of disorders related to lead exposure (76). An epidemiological study of the male reproductive system has demonstrated positive correlations between seminal plasma lead and spermatozoa ROS levels (77). On the other hand, in people with protracted exposure to lead, increased activity of superoxide dismutase has been observed, which suggests an adaptive mechanism against the increased amount of ROS production induced by lead (78). This may result in oxidative cell in the damage in reproductive tissues closely associated with ROS production. For example, a study on rat sperm exposed to ROS *in vitro* has demonstrated premature acrosome reactions and reduced penetration rate in the zona-intact (79). However, from low to high doses, there are known to be different responses of lead-induced oxidative stress in various target sites, including sperm (80). Studies on lead-exposed rats have demonstrated that lead influences sperm function, decreases serum testosterone levels and produces early onset of capacitation by activating pathways of ROS generation (32, 81). Additional evidence where rats were chronically exposed to lead has reported an elevation in the concentration of lipid peroxide in reproductive organs (82). Results of studies suggest therefore, that lead-induced ROS is an important molecular mechanism for male reproductive disorders, either in the hormonal stages or during spermatogenesis. 

## Conclusion

Examination of experimental data from both epidemiological and animal research suggests that lead in different concentrations has a wide spectrum of toxicity on the male reproductive system, including spermatogenesis, sperm functional parameters and reproductive hormones. Although unfavorable reproductive effects usually occur at relatively high levels of lead exposure (83), lower doses for longer time periods may also alter the male reproductive system in a manner similar to that previously reported at higher doses for shorter periods (58). Furthermore, regarding dosage level and duration of exposure, there are other potential factors to consider such as individual differences, social conditions (84), and various environmental factors. As a result of these numerous, potential confounders, it has not been easy nor straightforward to quantify which organ or pathways are involve in lead’s adverse effects on male fecundity. 

Although, reproductive tissues represent one critically sensitive organs to toxic substances such as lead, some studies have actually failed to demonstrate significant correlations between increased lead concentrations and impairment of the gonads. Additionally, low lead concentrations in the testis, seminal fluid, and epididymis (85, 86) have demonstrated that the blood-testis barrier may protect testicular cells from direct exposed to the high levels of blood lead. As such, there might be other pathways which reduce spermatogenesis among lead-exposed males. Or, on the other hand, spermatogenesis disorders are not completely predictable. 

Thus, according to wide spectrum effects of lead at different concentrations on reproductive hormones and the priority of hormones for growth, development, and function of the sex organs and spermatogenesis, the present review suggests that lead’s effects the male reproductive system most likely by disrupting hormonal regulations, mostly via the HPT axis, then reduces sperm production in seminiferous tubules of the testes.

The present review of lead toxicity on the male reproductive system also suggests that hormonal disruption might occur at lower levels of blood lead. Despite such findings, it has not been easy to definitively ascertain the correlation between lead exposure, male fecundity and probable mechanisms of infertility. For these reasons and perhaps because high levels of lead exposure might affect many organs in various ways, aside from the reproductive system, contemporary research has begun to shift more towards studies of low level exposures-particular with blood lead concentrations below the currently accepted worker protection criteria (<10μg/dl) as they may still adversely affect male fertility (87). 

As such, occupational health surveillance must continue to include the assessment of adverse effects on the reproductive system of lead-exposed workers, particularly those with significant environmental exposures. Because laboratory findings cannot definitively ascertain fertility status, this review suggests that future studies should aim to establish more concrete links between lead’s effects on reproductive dysfunctions and reduced fertility rates; not only changes in hormonal or sperm characteristics among lead-exposed subjects.
